# Weighing Costs and Benefits of Delay and the Acceptance of Two Decision Support Tools in Mental Health Care: Scoping Study Using Quantitative and Qualitative Data

**DOI:** 10.2196/71678

**Published:** 2025-09-30

**Authors:** Sarah McKenna, Min K Chong, Adam Poulsen, Ashlee Turner, Carla Gorban, Jacob J Crouse, William Capon, Mathew Varidel, Melissa Aji, Elizabeth M Scott, Ian B Hickie, Frank Iorfino

**Affiliations:** 1 Brain and Mind Centre, Faculty of Medicine and Health, University of Sydney, 94 Mallett St, Camperdown, NSW, 2050, Australia, 61 0419517723

**Keywords:** youth mental health, digital mental health, youth, adolescence, technologies

## Abstract

**Background:**

Mental disorders are the leading cause of disability in young people (aged 12‐30 years), and their incidence constitutes a major health crisis. Primary youth mental health services are struggling to keep up due to overwhelming demand, the complexity and severity of young people presenting for care, and a shortage of qualified mental health professionals (MHPs). Artificial intelligence (AI) tools have the potential to facilitate necessary improvements to diagnosis, triage, and care planning for young people with emerging mental disorders.

**Objective:**

The objective of the present scoping research was to examine beliefs and attitudes underlying MHP acceptance of AI tools in youth mental health services.

**Methods:**

In total, 57 MHPs (mean age 35.35, SD = 9.50 years, 72% female (n = 39)) with experience working with youth populations (age 12-30) took part in study 1 that involved completing a web-based survey about the acceptability of using AI in early intervention services. During study 2, 15 MHPs also participated in 1-hour semistructured Zoom interviews. Attitudes toward the use of 2 novel AI prototypes (both of which provide recommendations for care coordination based on previously published data analyses) in youth mental health were explored. Quantitative data were interpreted using descriptive statistics, and qualitative analysis followed the thematic analysis approach.

**Results:**

MHPs were more likely to agree than disagree that AI will improve youth mental health care overall (eg, n=37, 64% participants somewhat or strongly agree that the field of mental health will improve with AI). Despite voicing concerns regarding data security and privacy, MHPs also acknowledged a need for AI to improve the “signal-to-noise ratio” in services and address delays to care for those with severe and complex problems. Such problems were seen as pervasive across the youth mental health system and emphasize the serious costs of delaying the development and implementation of novel tools. All participating MHPs discussed the potential negative impacts of not adopting novel tools.

**Conclusions:**

MHP acceptance and uptake of novel AI tools in youth mental health services will be driven by a more complex cost-benefit analysis of both adopting and not adopting, rather than solely on their design. The costs of delay are clear, and so researchers and MHPs have a shared imperative to develop useful and meaningful clinical tools and to work jointly on integrating them into practice. Limitations of our sample (including low sample size limiting generalizability) notwithstanding, these findings should inform the future design and implementation of such tools.

## Introduction

Mental disorders have profound negative effects on young people’s health, well-being, and socio-occupational functioning, and the impacts typically continue throughout their life [1]. Mental disorders are the leading cause of disability among 10- to 24-year-olds and are the leading cause of death among people aged 15-44 in Australia [2-4]. In response to the extreme scale and complexity of these problems, urgent calls have been made to radically transform youth mental health services so that they are able to provide more efficient and personalized treatments [5,6]. For example, in Australia, this has led to the development of *headspace*, an early intervention youth mental health service that can provide multidisciplinary care to young people with emerging mental disorders [7,8]. Similar early intervention services have also emerged internationally, for example, in Canada, the UK, and Ireland, reflecting that youth mental health is currently a global health crisis [9-12]. Yet, these services are frequently overburdened and under-resourced, creating an urgent need to improve assessment and care coordination, particularly for those with emerging severe and complex problems [5,13,14].

To address this need, there is a rapidly growing interest in the development of artificial intelligence (AI) tools that can provide data-driven solutions to more efficiently and accurately plan appropriate treatments, though such tools are yet to be successfully implemented at scale in youth mental health services [15-21]. Research has shown that there are unique challenges to implementing AI tools in health care settings, including regulatory restrictions and clinical decision systems, along with a lack of trust as compared to other industries, likely because making a mistake in health care settings could have serious consequences, even death [22,23]. Even so, these perspectives fail to consider the potential benefits of improving decision-making systems, particularly in settings that are under-resourced and lack qualified staff. This points to a need for more targeted research focusing on specific challenges and opportunities for implementing unique tools based on the usefulness of the individual tool, characteristics of the setting, and broader contextual factors.

Various frameworks have been proposed to identify factors that may underpin successful implementation of novel technologies. The technology acceptance model (TAM) and the unified theory of acceptance and use of technology (UTAUT) are both widely accepted theoretical frameworks [24-27]. TAM proposes that technology acceptance is linked to perceived usefulness and perceived ease of use and is one of the most widely used and earliest theories in health care industries. UTAT is a broader framework that includes performance expectancy (how much a person believes that using the system will improve job performance) and effort expectancy (the perceived simplicity of using the system) along with cultural and organizational factors such as social expectancy (the perception that others believe the technology should be used) and facilitating conditions (technical or organizational support). Both frameworks are likely to have benefits for understanding this complex problem. While it is important to understand organizational- and system-level barriers, given that this is a unique context facing significant challenges, mental health professionals (MHPs) may also consider factors independent of organizational or social setting. For example, young people often present to mental health services with high levels of risk and low functioning, which may be linked to less trust in novel tools to assist with identifying severe clients, given the potential consequences of mistakes.

So far, there is limited research on the use of tools to assist with multidisciplinary assessment and care coordination in mental health care settings, particularly early intervention services, as current research tends to focus on the use of tools within therapy or as self-help tools [5,6,28]. Broadly, barriers to implementation among MHPs include low perceived usefulness of the tools, poor access to training and supervision, and poor perceived competence and knowledge in using clinical prediction models [29,30]. Additionally, therapist characteristics such as external feedback propensity (the degree to which someone values or is open to feedback) and feedback utilization (the extent to which someone implements feedback) significantly mediate the effectiveness of digital tools on client outcomes [31]. Given these barriers, it is important for design and implementation research to engage with youth MHPs to understand how their acceptance and uptake can be improved.

The present study focused on 2 tools that can be used by MHPs that are intended to improve triage and care coordination of young people with emerging mental disorders. Clients and MHPs can input information regarding clients’ multidimensional needs, which is then processed by algorithms developed from data on youth populations presenting to early intervention services. Tool 1 is focused on assessing the level of complexity so that clients can be triaged into short-term therapy or more high-intensity and multidisciplinary treatments [32]. Tool 2 is focused on predicting clients’ functional trajectory.[33] Those who have a high likelihood of deteriorating likely require more focused functional supports compared to care as usual. Currently, rough prototypes of these tools have been constructed as proof of concept (and so are not publicly available), and we sought MHP feedback to inform their ongoing design and improvement.

Accordingly, this research explores MHPs' acceptance of two decision-making support tools within a unique context, youth mental health services. The present paper describes 2 studies. Study 1 aimed to assess MHP attitudes toward the use of AI tools within youth mental health services more broadly, and the aim of Study 2 was to gain feedback on MHP acceptance of 2 novel AI tools that have been designed to improve assessment and treatment planning in youth mental health services.

## Methods

Two scoping studies were conducted separately, each with unique aims; quantitative and qualitative results were not integrated. Study 1 involved descriptive analyses of general attitudes toward digital tools amongst youth MHPs. Study 2 involved in-depth qualitative analysis of MHPs’ attitudes and beliefs. These research studies were not preregistered. Data from the study are available on request.

### Recruitment and Informed Consent

We used snowball sampling via social media advertisements (using LinkedIn and Facebook) and referrals from participants or other MHPs that had existing relationships with our team to recruit MHPs who had experience working with young people. MHPs completed a digital consent form and indicated whether they were willing to participate in a one-on-one interview, a survey, or both. Participants willing to participate in a Zoom interview were contacted via e-mail and were reimbursed for participating in both the interview and the web-based survey. Recruitment for surveys was time-limited as this was a scoping study that was intended to inform further development and testing of the digital tools; it was carried out between August 2023 and October 2024. Recruitment for qualitative interviews continued until saturation had been reached in April 2024. Participants were included in the study if they were health professionals who had provided mental health care to adolescents and young adults (12-30).

### Ethical Considerations

This study was approved by the University of Sydney’s Human Research Ethics Committee (2021/HE000680). Informed consent was obtained from all participants via web-based forms before participating. All data was deidentified before analyses and no identifying information was included in the current paper. Participants were compensated for completing virtual Zoom interviews.

### Study 1: Quantitative Data Collection

We first collected web-based survey data about clinicians’ acceptance of decision-support tools in youth mental health care to provide descriptive information about (1) the extent to which MHPs were likely to see digital tools as acceptable and useful, and (2) the potential barriers or facilitators of engagement over and above acceptance of the tools.

### Measures

#### Acceptance of AI-Powered Care Pathways

We adapted 8 items from an existing survey by Cornelissen et al [24] based on the UTAUT, a previous study demonstrated internal consistency and inter-rater reliability of the items, to assess what factors were likely to impact MHPs’ acceptance and uptake of AI tools in youth mental health services. Participants were asked to rate statements such as “I believe AI would be useful in my overall performance” on a 5-point scale from “strongly disagree” to “strongly agree.”

#### Potential Impact of Innovations in Artificial Intelligence

We used 10 items to assess MHP’s opinions about the potential of future AI to replace key tasks carried out in youth mental health services. These items were adapted from a survey by Doraiswamy et al [34] that assessed psychiatrists’ opinions on AI to be more appropriate for youth settings and a range of MHPs, including service managers, psychiatrists, psychologists, etc. The utility of these items has been established in previous studies [35,36]. Participants rated the potential importance of using AI to improve various tasks such as “treatment planning” and “identifying referrals to other services” on a 5-point scale from “not at all important” to “very important.”

#### Facilitators and Barriers to Engagement

We used 12 items to assess what attributes of novel tools and implementation strategies would most likely impact uptake of novel tools in youth mental health services. Items were adapted from a ranking exercise designed by Leigh et al [37] and were based on a literature search along with expert consensus. Example items included usability, an established research evidence base, and endorsement by colleagues. Participants were asked to rate attributes such as “usability” using a 5-point rating scale from “not important” to “very important.”

#### Procedure

We used web-based surveys to collect quantitative data on the acceptability of AI to health professionals in mental health settings and on the potential facilitators or barriers of implementation and engagement. We used descriptive statistics to compare responses to these items. Given the available sample size, it was not possible to analyze any differences in attitudes between various items. Before analysis, data were screened for valid responses (ie, reported job role fit inclusion criteria for study and location of workplace was in Australia). Given that we used descriptive statistics, participants with missing data were excluded from analyses.

### Study 2: Qualitative Data Collection

Participants were initially shown the digital tools and asked to provide initial impressions of the tool before commencing the interview. Semistructured interviews (see Multimedia Appendix 1) allowed us to gain more in-depth information about health professionals’ attitudes toward decision support tools within youth mental health settings. Qualitative data collection and analysis followed Standards for Reporting Qualitative Research [38].

#### Tool 1: Stratified Scoring of Young People’s Clinical Needs

Participants were shown a stratified scoring tool that had been embedded within the Innowell platform. The Innowell platform facilitates multidimensional assessments by allowing clients and their MHPs to complete in-depth initial assessments and then track progress during treatment in 5 key domains: (1) overall health, (2) mental health, (3) suicidal thoughts and behaviors, (4) everyday functioning, (5) social connectedness, and (6) drug and alcohol use. This platform has been described in detail in previous publications [39,40].

**Figure 1.**
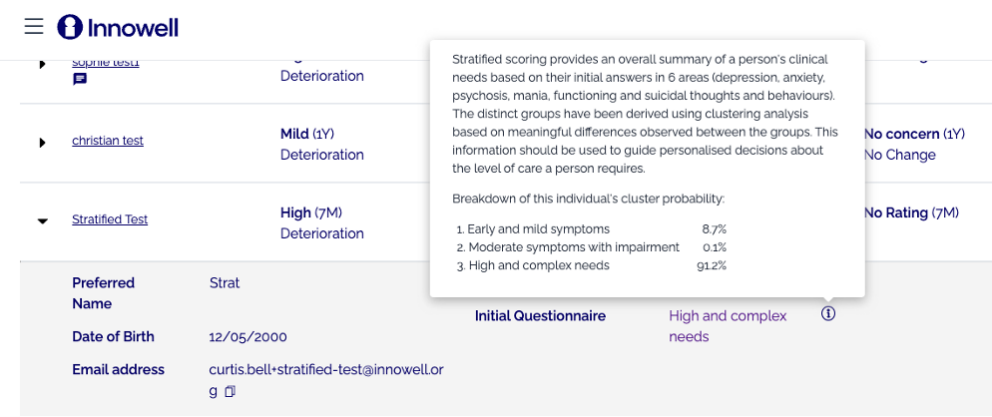
Depiction of tool 1 designed to provide stratified scoring of young people’s clinical needs.

As shown in Figure 1, the stratified scoring tool is designed to improve identification of subgroups who have complex multidimensional needs to determine appropriate service pathways and care options. MHPs are given an estimate of the probability that a client has (1) early and mild symptoms (low and mixed symptomatology with limited functional impairment), (2) moderate symptoms with impairment (established depressive symptoms and functional impairment), or (3) high and complex needs (very high and complex needs with functional impairment, suicidality, and at-risk mental states (psychosis or mania). The probabilities presented to MHPs are based on a latent class analysis of a cohort of 1284 people aged 12‐25 years presenting to youth mental health services, that has been described in more depth in a previous publication [32].

#### Tool 2: Predicted Functional Change

**Figure 2.**
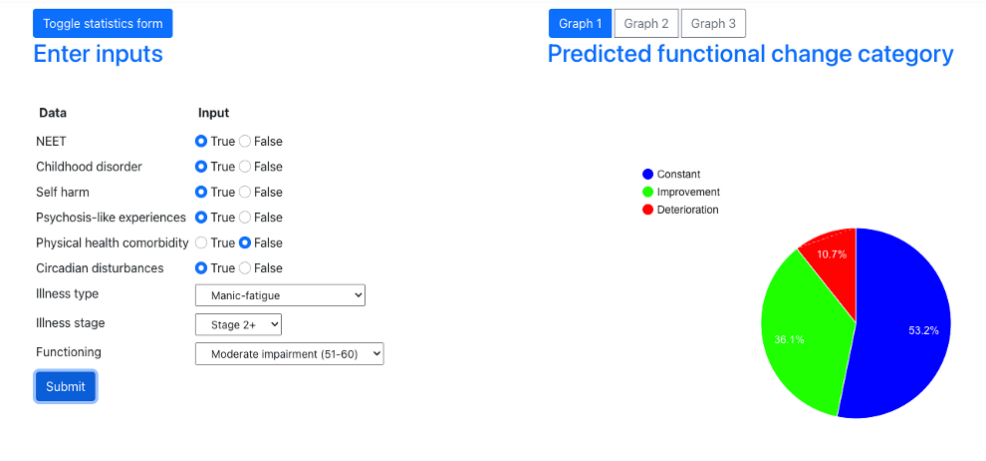
Depiction of tool 2 showing predicted functional change.

Participants were also shown a tool that predicts a client’s functional impairment trajectory over a 3-month period. As shown in Figure 2, the tool shows MHPs the likelihood that a client’s functional impairment will (1) remain constant, (2) improve, or (3) deteriorate. The prognostic model was developed from a sample of 718 young people (12‐25 y) who were engaged in youth mental health care, and it has been described in more detail in a previous publication [8]. In total, 8 factors were used for prediction, including employment, education or training status, self-harm, psychotic-like experiences, physical health comorbidity, childhood-onset syndrome, illness type, clinical stage, and circadian disturbance.

### Measures

We designed a semistructured interview based on components of TAM that aim to understand why users accept a given technology and how user acceptance can be improved through technology design. According to this framework, acceptance can be measured by behavioral intention to use a novel tool. Interview questions were focused on better understanding 4 key themes, including (1) first impressions of usability, (2) performance expectancy of the tool in decision-making, (3) predicted use within workflow, and (4) professional or ethical concerns.

### Procedure

Semistructured interviews were conducted via Zoom, video-recorded, and later transcribed. Interviews lasted approximately 60 minutes. Transcriptions of the interviews were analyzed using thematic analysis techniques, with the aim of establishing themes regarding the acceptability of our tools within youth mental health services [41]. Data analysis was both inductive (what was in the data) and deductive (informed by TAM) [24]. Two academic researchers (SM and MC) independently established a list of codes based on transcripts of the interviews. Subsequently, these codes were shared and discussed with a master coder (FI) to establish key themes. A second round of coding was conducted using a similar process to establish broader patterns of meaning within each theme and refined during a face-to-face meeting with the master coder. Trustworthiness of data coding was established by using two coders and a master coder for triangulation and through an audit trail that can be made available on request.

## Results

### Participants

The sample consisted of 57 MHPs (mean age 35.35, SD 9.50 years, 72% female (n = 39)) with experience working with youth populations. The majority of participants (45, 81%) came from metropolitan areas compared to rural or regional areas, with a median of 6‐10 years of clinical experience. In total, 21 participants (37%) had 10 years of experience or less, and 4 participants (7%) had 20+ years of experience. In total, 16 (28%) participants worked only in private settings, 17 participants (30%) worked only in community or hospital settings, while the remainder worked across a variety of settings. All participants completed the web-based survey, while 15 also agreed to participate in one-on-one semistructured interviews.

### Quantitative Data Analysis of MHP Acceptance of Novel AI Tools in Youth Mental Health Services

**Figure 3.**
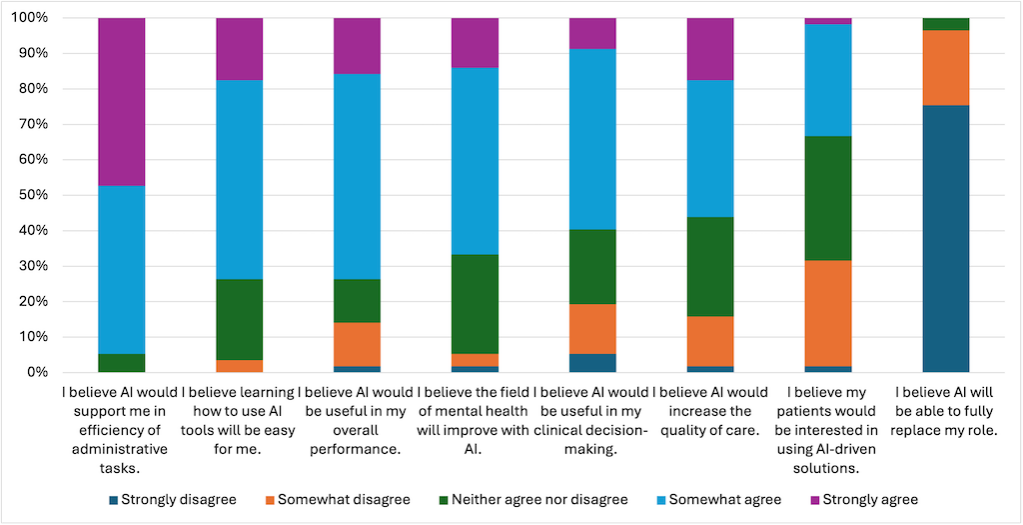
Levels of agreement among mental health professionals regarding the potential of AI to improve aspects of mental health care.

As shown in Figure 3, we first asked participants to rate the perceived usefulness of AI to improve clinical care. In general, participants were more likely to agree than disagree that AI (1) would benefit their overall performance, (n=40, 71%, strongly or somewhat agreed); (2) would improve the field of mental health, (n=37, 64% strongly or somewhat agreed); (3) would be useful in clinical decision-making, (n=33, 58%) strongly or somewhat agreed); and (4) would increase the quality of care (n=31, 54%) strongly or somewhat agreed. Almost all (n=52, 92%) participants strongly or somewhat agreed that AI would improve the efficiency of administrative tasks. Most participants (n=40, 71%) also agreed that AI tools would be easy for them to learn. Even so, participants were unsure whether clients were interested in AI-driven solutions, with (n=20; 34%) reporting they neither agree nor disagree that clients would be interested. Most participants (n=53, 93%) strongly or somewhat disagreed that AI would fully replace their roles.

**Figure 4.**
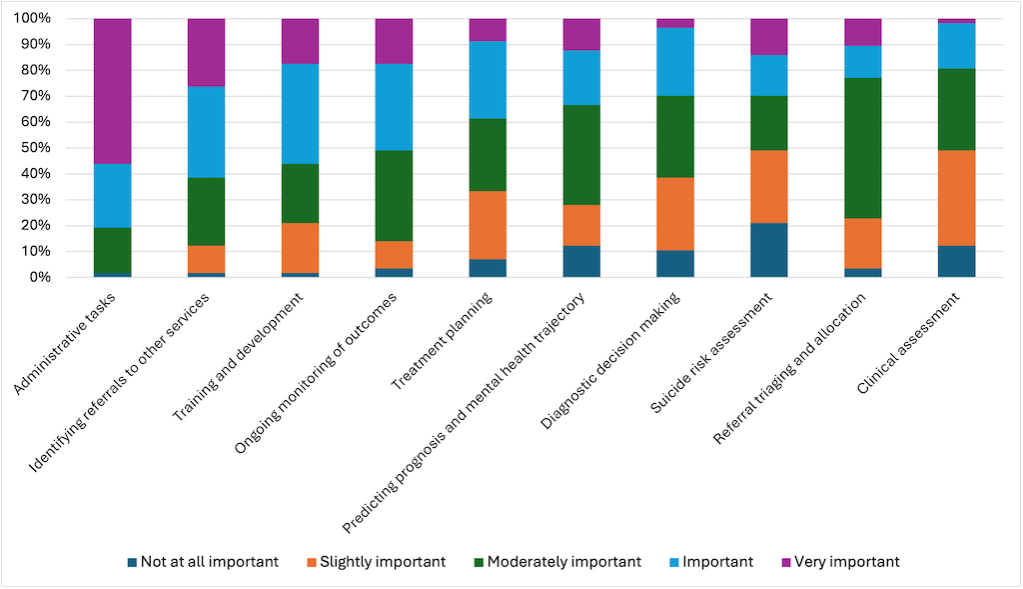
Perceived importance of using AI to improve various clinical tasks according to mental health professionals.

We also asked participants to rate the importance of using AI to improve various clinical tasks (see Figure 4). Participants were least likely to believe that AI was important for suicide risk assessment (42% (n = 24) rating it as not at all or slightly important) and clinical assessment (50% (n=29) rating it as not at all or slightly important) and were most likely to believe that it was important for improving administrative tasks (82% (n=47) rating this as very important or important). Over half (50% (n=29)) of participants believed it was important or very important for AI to improve ongoing monitoring of outcomes, identification of referrals to other services, and training and development. For other items, including referral triaging and allocation, clinical assessment, diagnostic decision-making, and predicting prognosis and mental health trajectory, participant ratings appeared to be more mixed.

**Figure 5.**
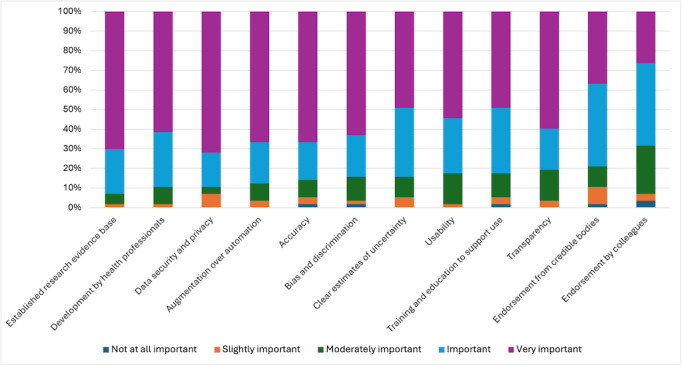
Perceived importance of various aspects of implementing and designing novel digital tools according to mental health professionals.

Finally, as shown in Figure 5, participants were asked to rate the level of importance of various attributes when considering implementing AI solutions into their practice. All items had high levels of agreement (ie, over 60% (n=34) agree or strongly agree). Over 90% (n=51) of participants saw an established research evidence base, development by health professionals, and data security and privacy as important or very important. Meanwhile, over 80% (n=46) of participants reported that accuracy, augmentation over automation, bias and discrimination, usability, endorsement from credible bodies, clear estimates of uncertainty, transparency, and training and education support were important or very important.

### Qualitative Feedback on AI Prototypes as Proof of Concept

Overall, we found that participating MHPs were highly interested in the development of AI tools. Thus, rather than simply focusing on the prototypes they were shown, discussions focused on the costs and benefits of adopting and not adopting diagnostic and prognostic AI tools in youth mental health services more generally. In line with survey results, participating MHPs were optimistic about the potential improvement to youth mental health care that could be achieved through AI. However, they also appeared to be highly cautious about potential risks to clients and about financial, legal, or training burdens on themselves.

### Costs and Benefits of Adopting Novel Tools in Youth Mental Health Services

#### New Tools Need to Overcome the Non-Negotiable Barriers to Adoption (Costs)

Data fluency was a key concern, as participants often struggled to interpret the results of Tool 1 (see Table 1, Theme 1.1). For example, participants were often surprised by the case study used to demonstrate Tool 1, as the individual was shown to have the greatest probability of being in the low- or high-risk group but no probability of being in the moderate group, which several participants interpreted as an error. One participant noted that for such tools to be implemented, MHPs would need to be well trained in more complex statistical models. By comparison, Tool 2, which used a pie chart to demonstrate predicted trajectories, was immediately seen as more “user friendly,” visually appealing, and easy to understand.

**Table 1.** Qualitative feedback on artificial intelligence (AI) prototypes from youth mental health professionals.

Themes	Sample excerpts from qualitative data
Costs and benefits of adopting novel AI tools	
Financial costs, time burdens, and safety concerns (costs)	
1.1 Training and other resources needed to understand complex algorithms and establish trustworthiness, reliability, and validity.	“… it’s based on probability, right, which is not deterministic, and it’s not likely chance. And so it’s something we have to get used to, in terms of things not being black and white.” [Participant #3, Male] “I don’t know if I’m smart enough to understand the algorithms. But I just, I’d be very interested in reading the, like the administration manual, or very dumbed down research paper that I could go, oh, this is why they gave that particular weighting… this was the clinical team and the evidence base that they drew upon.” [Participant #17, Male] “I want to know that it has been tested in clinical and community samples, you know, I want to know about its reliability and validity… all that sort of stuff.” [Participant #6, Female] “What one person thinks is complex at the beginning of their career, that language is imprecise between people. And so having something standardized and saying, well, 91.2% chance of this… I mean, that seems pretty helpful to me.” [Participant #19, Male]
1.2 Data security and privacy	“I’d want to know like how secure it is, like who can access this information?” [Participant #24, Male] “Oh, just feels really intrusive in the person’s life and in a way that I wouldn’t be comfortable with. So I think that’s one thing that’s put me off.” [Participant #15, Female]
1.3 Professional liability for decision-making	“If the clinician doesn’t look at it, kind of like reading a doctor’s referral letter, and they miss something, will you be liable?... you should probably be liable, because you’re not looking at important information… like that is a professional issue… And on the flip side, if you make a clinical decision or a service decision that is maybe counter to the indications… say there’s a coroner’s inquest and they say, well, the tool said actually you should have referred and why didn’t you?” [Participant #18, Male]
1.4 Ethical concerns about sharing predictive data with clients that may lead to helplessness or reduced self-efficacy.	“I think for people that maybe don’t have that sort of level of insight… about their own functioning or their own prognosis… this would be really shocking for them… with someone that was like, oh, yeah, you know… there’s nothing wrong with me, or this isn’t my fault, or any of those sort of, like core beliefs about what’s going on for them. I can imagine them seeing this would be really sort of shocking, or really disarming.” [Participant #17, Male] *“*People feel quite heard and seen by completing these things. And I think that’s something if, if that can be made front and centre of these measures… clinicians have their own biases about X, Y, Z, but actually the client experiences, it’s helpful… even clients being told that I think that, yeah, helpful message.” [Participant #9, Female]
1.5 Financial cost and time burden of implementing novel tool	“Some of the clinicians would be like, I don’t have time to… look at all that data before I see someone... And so I can see that that’ll be a struggle, like how do you make this really quickly accessible as well.” [Participant #5, Female] “Depends about the cost… if it was free, and I knew the data wasn’t going to go anywhere, and it was just for me and my practice, I’d use it every day. Okay. If it costs me money, depending on how much… I’d probably say a two out of ten [likelihood of using]. Okay. And if I’m desperate with a complex case… I would, you know, I’d give it like a seven out of ten... If it was a really small fee, I would give it to all my clients prior to them starting.” [Participant #18, Male]
*Expected use for tools within existing workflows (benefits*)	
2.1 Faster identification and referrals of those with severe and complex needs—particularly in large multidisciplinary clinics	“I think it would be very helpful… there have been instances where we have had a few clients come in, and we just weren’t the right service for them… emerging psychosis or things like that, which were just not the place for. So I think this would just be so helpful in being able to identify that early on.” [Participant #24, Male] “If I ran the headspace center still… I’d be like an eight out of ten [chance of adopting], like working really hard to see how we could bring this into the center.” [Participant #19, Male]
2.2 Can supplement rather than replace clinical judgment, which is naturally biased, and dependent on people’s training and experience	“Clinical decision making and clinical judgment, has a pride of place… there’s kind of entrenched deep problems with that, your biases… I think the clinician has to be pretty open… the statistical tool is probably more accurate than my feeling that someone is suicidal or not… If the test results are coming back and saying 86% chance of being cancer, you should probably agree with that rather than like, your various biases based on your clinical experiences that have selective memories and things like that.” [Participant #5, Female]
2.3 Can reduce burden of identifying evidence-based treatments particularly for early MHPs assessing severe and complex clients	“It would be great to have something that could generate a type of like rudimentary treatment plan… your client is presenting with all their history, all this information, and it could like, all the latest research and intervention… that would save so much time, because as a clinician, I think that’s where so much of my time is taken just like treatment planning and stuff.” [Participant #16, Female] “Seeing what’s the best intervention out there, perhaps as a space where AI can help. Okay, ADHD, the first line of treatment is CBT… gathering all the research that’s effective there…” [Participant #18, Male]
2.4 “What gets measured gets done”—better information for funders and governments regarding population needs	“Whatever gets measured, gets done…. you get funded by literally the occasions of service you do… if you had this broader sense of, you know, the social occupational functioning and depression, anxiety, and maybe some other things to consider, like if that was all data that you could report back on, to your funders to say this is the targets that we’re about… I really like that idea too.” [Participant #4, Male] “I think it’s really helpful from a service perspective… to look at how they allocate their resources, but it would need some change… you know, are they seeing people [with] early symptoms, are they mostly moderate, you know, what are the what are the breakdown, and people are presenting?” [Participant #9, Female]
2.5 “Clinicians will do what they always do”	“They just still continue to do what they do, they don’t necessarily take all that information into account as we know… actually feeds back into people’s skills and education.” [Participant #5, Female] “I don’t know that individual clinician workers actually, necessarily then take that into account in terms of treatment planning. They’ve ticked off the assessment that’s done and then the person is allocated according to what the service can actually provide, not necessarily what the young person needs.” [Participant #2, Female] “I think it’s up to how much the clinician uses the information again, if a clinician is sensitive to what this data is, is telling them, then I think it can have a significant impact on client outcome. But yeah, I guess, you know, therapy needs to change as a result of this to impact the outcome.” [Participant #19, Male]
Costs and benefits of not adopting AI tools	
Irreplaceability of the “human touch” (benefits)	
3.1 Loss of empathy, understanding, and connection	“This is a real person answering the question. There must be some other issues here… physical, family, and help seeking history to complement what’s been responded to - the why.” [Participant #15, Female] “The AI is not a human being with a culture with understanding and maybe empathy. Of course, we could build it in, we could build it into the AI, but again, you know, at what cost does that have? Like, what risk is that? You know, would I be comfortable speaking to an AI? I don’t know…” [Participant #16, Female]
3.2 Skeptical that treatment progress can be predicted by algorithms.	“So I guess I will check you know, if I if I agree with that statistic. And if I, if I did think that too, then I would look at you know, maybe you know, getting them more supports or connected to different resources…” [Participant #9, Female] “I think because improvement in mental health is so variable… client can have really different goals to begin with, goals can change throughout with treatment… it’s never linear improvement either… like you might not know at the start of treatment that they are on the spectrum and so you could be trying to treat them as such, but it’s like, then they’re not going to demonstrate improvement because there’s that crucial detail that you missed.” [Participant #10, Female]
3.3 MHPs pref their own clinical judgment	“This person’s needs haven’t been understood through this data. It’s just their symptoms have been understood… So there’s a need to be really cautious about… how we’re presenting the data, I guess, and what we’re saying the data can and can’t do.” [Participant #6, Female] “I could see its potential benefits, but… there’s a part of me that’s like… it can never override human judgment… it’s helpful, but I guess it’s not the be all and end all. That’s just my initial sort of reaction… I guess it would sound like a tool that I have to help support my decision making… it’s still up to me to decipher and make sense of the information and to come to my own conclusion about it.” [Participant #10, Female]
3.4 “Clinicians will do what they always do”	“They just still continue to do what they do, they don’t necessarily take all that information into account as we know… actually feeds back into people’s skills and education.” [Participant #5, Female] “I don’t know that individual clinician workers actually, necessarily then take that into account in terms of treatment planning. They’ve ticked off the assessment that’s done and then the person is allocated according to what the service can actually provide, not necessarily what the young person needs.” [Participant #3, Male] “I think it’s up to how much the clinician uses the information again, if a clinician is sensitive to what this data is, is telling them, then I think it can have a significant impact on client outcome. But yeah, I guess, you know, therapy needs to change as a result of this to impact the outcome.” [Participant #15, Female]
*The hidden costs of delay (costs*)	
4.1 High demand and high complexity in primary youth mental health services along with “skills shortage” leading to delayed care and referrals	“We have a huge demand for services, we have people presenting with very mixed symptoms, we have them coming into systems where there is very low levels of expertise… I see AI as being a tool which will help to help to improve the signal to noise ratio, will help to be able to discriminate quickly, those people who need to be stepped up into a kind of higher level of care, to more intensive care or monitoring, and those people who can be treated with a kind of briefer, less intense kind of treatment, and they can be triaged accordingly, and then kind of monitored and tracked accordingly.” [Participant #5, Female “I think it would be helpful when I’m a bit uncertain about a client in terms of how complex I feel like it is going to be… and whether I’m feeling like, they might need a different service, potentially. Kind of having a bit of a clear line of why we might be referring them on.” [Participant #4, Male]
4.2 Service managers and supervisors have poor oversight of less experienced clinicians and not involved in intake assessments	“If you have people with different levels of training and different theoretical models and so on, I think it’d be helpful… that shared data… I could see it being helpful in a supervision setting, too, because it’s sort of more objective data that you can bring into the supervisee supervisory relationship, and discuss and reflect on…” [Participant #8, Female] “A really, really clear example I can think of is if there’s someone who is reporting psychotic, like experiences and it’s a less experienced clinician, that person is going to be more inclined to go or shit they’ve got psychosis, I need to send them to hospital as opposed to… what is it actually based on? … as that support tool, as an adjunct for that clinical experience.” [Participant #4, Male]
4.3 Moral obligation to clients to adapt and improve.	“I think the input we can provide is making sure it’s reliable, valid, and so on. But when it comes to preferences, it’s not really my own preference… it’s really what the folks in front of us want to utilize. And so we need to be open minded to that and not biased.” [Participant #17, Male] “If these tools are improving, like health care outcomes, and are more accessible to people… it’s about trying to kind of reduce human suffering, like why would you really want to get in the way of that…. if you kind of imagine it in medicine, and they came up with some revolution, yeah, to kind of detect cancer or something, and then doctors, cancer specialists were like, ‘Oh, we don’t want to do that’ … we’d be up in arms about that sort of thing.” [Participant #24, Male]

Additionally, almost all users wanted detailed information about how complexity and functioning had been assessed, including understanding psychometric properties of assessment tools, seeing individual items that had led to a particular rating, and having a good understanding of the populations from which the ratings had been derived. Notably, however, improved training for assessing and identifying severe and complex clients was seen as an important gap, with or without novel tools. One MHP commented that having tools such as this may lead to a more uniform definition of complexity across health services and professionals, which could improve ease of referrals and care coordination.

Participating MHPs also raised concerns about security and privacy (Theme 1.2) and wanted clear information about who would have access to client data before using these tools. In addition, participants had concerns about professional liability if clients received inappropriate care or if risk was not responded to in a timely fashion because of either ignoring or following the advice of tools (Theme 1.3). Several participants reflected on the need for clear guidelines from professional bodies, as well as changes to legislation and insurance policies, before the implementation of these tools could become widespread.

Moreover, when discussing Tool 2, several participants discussed ethical concerns about sharing information about a client’s predicted trajectories with clients themselves (Theme 1.4). Participants were particularly concerned about the potentially damaging impacts on a client’s hope for recovery, their self-efficacy, and their motivation to continue with treatment. On the other hand, more experienced MHPs were more likely to see this tool as empowering, with one participant noting that they had used a similar tool in their practice and found that “people feel quite heard and seen by completing these things.”

Finally, the financial and time burdens of adopting novel tools within health services were frequently cited as a major barrier to implementation (Theme 1.5). Participants suggested that MHPs would only pay a fee for novel technology if they could quickly and easily understand the value of the tool.

#### Novel Tools Can Address Knowledge Gaps and Will Have Particular Benefits for Severe and Complex Presentations (Benefits)

Participating MHPs generally expected both tools to perform well as a “red flag” system, particularly in large multidisciplinary clinics with high demand for service, to improve efficiency of care coordination, and to help identify those with complex needs more quickly and easily (see Table 1, Theme 2.1). For this reason, participants who worked in private practice were less likely to see the value in the tools, given they did not generally triage clients before seeing them for initial assessments. However, several participants reflected on the difficulty of referring clients within the Australian “Medicare system” and the value of these tools for improving communication with general practitioners and other health professionals.

Despite concerns about the tools’ accuracy and reliability, several participants noted the value of having another source of information. They thought the tools could provide valuable insight about complex clients who were not responding to current treatment by assessing whether their current trajectory aligned with expectations (Theme 2.2). Participating MHPs reflected that no tools should be used in isolation; instead, they recommended that such tools should be “triangulated” with other assessment methods and their clinical expertise.

Several participating MHPs suggested that both tools could be improved by providing treatment recommendations, particularly to reduce the burden of care coordination for complex or unique presentations that may require a higher level of training and expertise (Theme 2.3). In a similar vein, participants also suggested that they would prefer these tools to help them identify a client’s needs from treatment (ie, need for functional supports), rather than diagnostic information, as this was seen as more relevant for care planning.

As well as helping individual clients to get more efficient care, one participant noted that “what gets measured gets done,” suggesting that if tools such as this are implemented in services, it will help funders to better understand client needs and support more diverse support options in youth mental health services other than individual therapy (Theme 2.4).

An important caveat is that participants predicted these benefits would not be realized without adequate training and support for participating MHPs. One participant commented that a lack of experience and expertise in treating severe or complex problems would limit the tools’ usefulness (Theme 2.5). They also predicted that the use of the tools would be limited by participants’ training in multidisciplinary care more broadly. Indeed, several participating MHPs working in private psychology practices commented that they did not directly address client functioning and did not see Tool 2 as relevant to them.

### Costs and Benefits of “Business as Usual” in Youth Mental Health Services

#### Essential to Preserve Benefits of the “Human Touch” (Benefits)

Even among participants who were highly open to these tools and believed they could be beneficial, there were significant concerns about replacing in-person assessments with digital tools (see Table 1, Theme 3.1). For the most part, participants were worried that digital tools could not display empathy and understanding or build connections with clients. They were also concerned that tools did not consider important contextual information such as family history, social support, or interpersonal communication styles. Additionally, participants were highly skeptical about tools predicting the likelihood of future trajectories (Theme 3.2), as they viewed progress in therapy as highly variable and influenced by a complex range of factors. Most reported that they would mainly use prediction tools retrospectively to assess whether treatments were achieving the intended effects or needed adjustments.

A related problem was that participants ultimately trusted their clinical judgment over and above algorithms to make final decisions about treatment planning (Theme 3.3). When asked, most participants stated they would trust their clinical judgment if they disagreed with the tools. Having said this, 3 clinical supervisor participants wanted to use these tools with less experienced MHPs to help them recognize biases in their thinking. These participants were also more likely to see “disagreements” with the tools as an important source of information that would enable more reflection and assessment, rather than seeing tool ratings as “right” or “wrong.”

#### Urgent Need to Improve the Signal-to-Noise Ratio in Youth Mental Health Services, and MHPs Cannot Get in the Way of Progress (Costs)

Importantly, most participants also wanted to discuss broader issues in youth mental health services that required urgent improvement through tools such as those used in the present study, indicating that whilst they had concerns about adopting novel tools, they also had concerns about business as usual. Participants frequently commented on issues such as “skills shortage” and the current high demand for services in Australian youth mental health services (see Table 1, Theme 4.1). Participating service managers were concerned that young people were presenting to care earlier in the course of illness (when symptoms are more mixed rather than meeting criteria for specific disorders) and in higher numbers and reported a shortage of skilled health professionals in Australian early intervention services. As such, the tools were seen to improve the “signal-to-noise ratio” within health services by identifying those with more severe disorders earlier and providing more efficient referrals and care coordination to appropriate services.

Additionally, 2 participating service managers cited occasions when clients had been turned away, despite presenting with concerning symptoms, due to lack of knowledge of intake staff (Theme 4.2). For this reason, they frequently discussed how our tools could improve supervision and oversight of less experienced MHPs in their services, particularly those making decisions about referrals and allocation of care.

There were also ethical and moral concerns about “gate-keeping” a client’s access to more accurate and comprehensive assessments (Theme 4.3). Two participants made comparisons to medical fields, such as cancer treatment, suggesting that if professionals in these fields were reluctant to adopt evidence-based tools, it would be seen as highly unethical. Participants reflected that the decision to use or not use novel tools should involve clients, particularly if they have been shown to improve outcomes.

## Discussion

In sum, MHPs that participated in our study were optimistic that AI would lead to improvements in youth mental health care overall. Despite non-negotiable barriers to adoption, including the financial and cost burdens of learning novel tools, participants found the tools to be useful “red flag” systems that could drastically improve the efficiency of referral and treatment planning processes. These findings highlight both the expected costs and potential benefits of adopting novel tools in clinical settings. Equally, all participating MHPs discussed the impacts of not adopting novel tools (despite not being asked directly), demonstrating the complexity of this decision. Interpersonal relationships with MHPs have important benefits for clients that should be protected. Even so, there are significant risks to clients if youth mental health services do not adopt more intelligent systems that can address the “signal-to-noise” ratio and ensure complex clients receive high-quality care faster.

This work provides a better understanding of how MHPs will make decisions about accepting and adopting novel AI into practice. Digital mental health research has reported consistent problems with widespread implementation of novel tools in youth mental health, partly due to MHP resistance [15-21]. Even so, MHPs in our study clearly saw the utility and value of AI (quantitatively and qualitatively), and this value ranges from reducing administrative burden to helping with referrals, prediction, and treatment planning. These benefits notwithstanding, MHPs identified serious challenges to overcome, including training requirements, infrastructure limitations, and professional liability concerns. Meanwhile, many of the barriers identified are highly solvable—not just in the future, but they must be addressed before widespread adoption can occur. If these barriers are addressed and benefits realized, MHPs clearly acknowledge the value of improved workflows, particularly in high-volume clinics, and predict important benefits for broader mental health system demand. This has the potential to raise the quality of decisions about evidence-based treatments by supporting them with quality information and predictions. Therefore, the costs of delay are clear. Researchers and MHPs have a shared imperative to develop useful and meaningful clinical tools and to work jointly on integrating them into practice.

These findings also demonstrate feasible avenues for improving MHP acceptance and uptake of novel tools. This study identified that usability, development by health professionals, an evidence base, and augmentation over automation are crucial. Again, many of these steps are highly achievable and have been successfully trialed. For example, previous research has found that health professionals are more likely to adopt AI tools when they have seen experienced colleagues integrate them into care and demonstrate potential use [24,31]. Comprehensive literature reviews have also established key steps that may be needed to reduce algorithm avoidance in health care, such as decision autonomy and incentivization [42]. Our results suggest the design of future AI tools should ensure that prognostic information is clearly linked to treatment recommendations and is framed as a means of supporting care planning (eg, by allowing MHPs to assess whether clients are progressing as expected), rather than being seen as deterministic. Given these steps are highly achievable, delaying rapid development and implementation of such tools would be a missed opportunity. There is an urgent need for researchers and MHPs to work together on redesigning current models of care, moving beyond ‘business as usual’ and toward innovative approaches that better address the complexities of mental health service demand.

Despite these contributions, our research also has limitations that should be addressed. Only 49 MHPs completed our web-based survey. This may reflect that participants who were willing to complete web-based surveys were more interested in AI and therefore more likely to be optimistic about potential future contributions. We also used snowball sampling that may have introduced bias by capturing participants with similar backgrounds and attitudes. Taken together, this limits the generalizability of our findings, and significant further research is needed amongst youth MHPs. We plan to address this in the future through more comprehensive research in real-world settings on the acceptability, feasibility, and usefulness of our tools. Moreover, we were unable to conduct subgroup analyses with our quantitative data that may help to explain whether AI acceptance is likely to vary based on professional background, experience level, or setting, or whether certain implementation strategies were seen as more valuable. Another limitation is that participants were shown prototypes and were not able to use the tools independently. Survey data suggests that MHPs are overly optimistic about the ease of understanding AI algorithms and so may have overestimated the usability and interpretability of the tools without support from the interviewer. For example, in survey items, MHPs were likely to agree that it would be easy for them to learn novel AI; however, we found that our prototypes were complicated for MHPs to understand, partly because they provided estimates of uncertainty rather than “black and white” information about client status. Addressing both limitations in future research should involve implementing prototypes in youth mental health services as early as possible, to gain more nuanced and diverse opinions from real-world settings.

In conclusion, MHPs recognize the potential of novel AI tools to solve urgent problems in youth mental health. While there are clear and non-negotiable barriers to adoption—including financial costs, potential liability, and training needs—MHPs consider these against the costs of delaying innovation. Problems accessing quality care, particularly for those with severe and complex problems, are pervasive across youth mental health services, creating a need to better handle “signal-to-noise” and to improve efficiency of treatment planning and referral processes. Future research can support MHPs by rapidly developing and implementing novel tools and helping integrate such tools into practice.

## Supplementary material

10.2196/71678 Multimedia Appendix 1 Semistructured interview guide for mental health professionals.

## Abbreviations

AI: artificial intelligence
MPH: mental health professional
TAM: technology acceptance model
UTAUT: unified theory of acceptance and use of technology
