# Supplementary material for: Weighing Costs and Benefits of Delay and the Acceptance of Two Decision Support Tools in Mental Health Care: Scoping Study Using Quantitative and Qualitative Data
Source: JMIR Hum Factors. 2025 Sep 30;12:e71678. doi: 10.2196/71678 (PMC12483475; doi:10.2196/71678)
Supplement: Multimedia Appendix 1 [file humanfactors-v12-e71678-s001.docx]

**Appendix A**

***Semi Structured Interview Guide – Clinicians***

Thank you for participating in this research project on artificial intelligence. We would like to record this interview so that your thoughts are accurately represented. There are no right or wrong answers and you may stop the recording at any time during the interview. I will also be taking a few notes during the interview. Is it okay if we proceed?

I’m going to be asking you some questions related to artificial intelligence and clinical decision support systems

| Questions | Prompts | Rationale |
| --- | --- | --- |
| What do you think of when I say “clinical decision support system” (CDSS)? |  | Warm up question  Gauge knowledge of clinical decision support systems |
| *Interviewer to present clinical vignette*  *eg 24 year old male with depression on a background of substance use etc…*  What are some decisions you would need to make in the context of treating this case?  How do you identify which problems to focus on?  How would you develop a treatment plan? What factors would you consider?  How do you make decisions about what to do about problems?  How would you make a decision about whether treatment is working or not? |  | Ascertain factors involved in clinical decision making processes |
| In the context of the current study, we define clinical decision support systems as a type of artificial intelligence system that analyse patient data and provide support to healthcare professionals to make decisions. This may involve providing advice based on clinically relevant guidance, recommendations, alerts or other clinical decision aids.  At the Brain and Mind Centre, we are in the process of developing a clinical decision support system. Using patient data, this tool provides a personalised prediction of outcomes such as functioning and suicidal ideation.  *Interview briefly shows them how the system works*  *Interviewer invites them to think out loud as they use the system*  **Think aloud prompts:**  What do you think you need to do here?  What do you think this means?  What do you think this is showing you?  What was your opinion on that?  How could this be improved?  What did you like/not like about that? | | |
| How was your experience of using the tool? | What did you like?  What did you dislike?  What do you see as the biggest challenges in using this tool?  What concerns would you have about the predictions?  How important is the | Gauging broad experience and attitudes towards tool |
| How would you feel about integrating the described technological CDSS into your daily clinical work? | How would it impact your clinical practice?  What would be the benefits? Disadvantages?  What features would be important to include?  Where would you not rely on AI? | Gauging attitudes towards the use of CDSS in clinical work |
| How would the tool impact your clinical decision making? | What would you do if there was a different between your opinion and the decision provided by the tool?  What would you do differently if there was a significant declining trajectory?  Would you share the data with your patient? | Gauging the clinicians perceived performance expectancy of the tool in decision making |
| When in the course of your clinical workflow would you want to see this information? | For example, in advance before an appointment (day before), morning of clinic day, during session, post-session  Why do you think it would fit best then? | Exploring when the clinician thinks this tool would be used within the clinical workflow |
| What are some possible scenarios where the tool would be most helpful and least helpful? | Specific types of patients  Specific moments in workflow/patient interactions  Specific settings  Why do you think it would be helpful/unhelpful in that particular scenario? | Exploring the clinicians perceived use of tool |
| What are the ethical or professional issues that may come up in using this tool in clinical practice? | For example, use of data, over reliance, bias | Exploring ethical/professional considerations |
| Can you tell me about your experience with any other clinical decision support system? | Which ones do you use?  Why did you start using them?  What do you think of them?  What do you like about them?  What do you dislike about them?  If no experience*:* why do you think you’ve never been exposed to CDSS? | Gauging extent of previous experience of CDSS |
| Based on the vignette, how would this tool impact your previous decisions? | How does this tool impact your predictions on the prognosis? Treatment plan? | Perceived impact of tool on clinical decision making |
